# Trajectory of Unawareness of Memory Decline in Individuals With Autosomal Dominant Alzheimer Disease

**DOI:** 10.1001/jamanetworkopen.2020.27472

**Published:** 2020-12-02

**Authors:** Patrizia Vannini, Bernard J. Hanseeuw, Jennifer R. Gatchel, Sietske A. M. Sikkes, Diana Alzate, Yesica Zuluaga, Sonia Moreno, Luis Mendez, Ana Baena, Paula Ospina-Lopera, Victoria Tirado, Eliana Henao, Natalia Acosta-Baena, Margarita Giraldo, Francisco Lopera, Yakeel T. Quiroz

**Affiliations:** 1 Department of Radiology, Athinoula A. Martinos Center for Biomedical Imaging, Charlestown, Massachusetts; 2 Department of Neurology, Massachusetts General Hospital, Boston; 3 Department of Neurology, Brigham and Women’s Hospital, Boston, Massachusetts; 4 Harvard Medical School, Boston, Massachusetts; 5 Department of Radiology, Harvard Medical School, Boston, Massachusetts; 6 Neurology Department, Cliniques Universitaires Saint-Luc, Brussels, Belgium; 7 Institute of Neuroscience, Université Catholique de Louvain, Brussels, Belgium; 8 Department of Psychiatry, Massachusetts General Hospital, Boston; 9 Department of Psychiatry, McLean Hospital, Belmont, Massachusetts; 10 Amsterdam University Medical Centers, Alzheimer Center Amsterdam, Amsterdam, the Netherlands; 11 Grupo de Neurociencias de Antioquia, School of Medicine, Universidad de Antioquia, Medellin, Colombia; 12 Department of Radiology, Hospital Pablo Tobón, Uribe, Medellin, Colombia

## Abstract

**Importance:**

Recent studies have suggested that unawareness, or anosognosia, of memory decline is present in predementia stages of Alzheimer disease (AD) and may serve as an early symptomatic indicator of AD.

**Objective:**

To investigate the evolution of anosognosia of memory decline in individuals who carry the *PSEN1* E280A variant for autosomal dominant AD compared with family members who do not carry the variant.

**Design, Setting, and Participants:**

This cohort study investigated a total of 2379 members of a Colombian kindred with autosomal dominant AD who were part of the Alzheimer’s Prevention Initiative Registry. Assessments were completed at the University of Antioquia, Colombia, with data collected between January 1, 2000, and July 31, 2019.

**Main Outcomes and Measures:**

Awareness of memory function was operationalized using the discrepancy between self-report and study partner report on a memory complaint scale. Linear mixed effects models were used to assess memory self-awareness over age separately in variant carriers and noncarriers.

**Results:**

This study included 396 variant carriers (mean [SD] age, 32.7 [11.9] years; 200 [50.5%] female), of whom 59 (14.9%) were cognitively impaired, and 1983 cognitively unimpaired noncarriers (mean [SD] age, 33.5 [12.5] years; 1129 [56.9%] female). The variant carriers demonstrated increased awareness until the mean (SD) age of 35.0 (2.0) years and had anosognosia at approximately 43 years of age, approximately 6 years before their estimated median age of dementia onset (49 years; 95% CI, 49-51 years). Cognitively unimpaired noncarriers reported more complaints than their study partners aged 20 and 60 years (10.1 points, *P* < .001). On the awareness index, a decrease with age (mean [SE] estimate, −0.04 [0.02] discrepant-points per years; *t* = −2.2; *P* = .03) in the noncarriers and in the variant carriers (mean [SE] estimate, −0.21 [0.04] discrepant-points per years; *t* = −5.1; *P* < .001) was observed.

**Conclusions and Relevance:**

In this cohort study, increased participant complaints were observed in both groups, suggesting that increased awareness of memory function was common and nonspecific to AD in this cohort. In variant carriers, awareness of memory function decreased in the predementia stages, reaching anosognosia close to the age of mild cognitive impairment onset, providing support for the usefulness of awareness of memory decline.

## Key Points

**Question:**

How does lack of awareness (anosognosia) of memory impairment evolve in the Alzheimer disease (AD) trajectory?

**Findings:**

In this cohort study of 2379 members of the Alzheimer’s Prevention Initiative Registry with a presenilin (*PSEN1* E280A) variant for autosomal dominant AD, awareness of memory function was increased in carriers and noncarriers approximately 14 years before their estimated median age of dementia onset (49 years). In variant carriers only, awareness of memory function was reduced in the predementia stages, reaching anosognosia 6 years before dementia onset.

**Meaning:**

The findings suggest that alteration of awareness of memory function in predementia stages may inform practitioners about AD progression.

## Teaser

This cohort study investigates the evolution of anosognosia of memory decline in individuals with the *PSEN1* E280A variant for autosomal dominant Alzheimer disease compared with family members without the variant.

## Introduction

Memory loss is a key feature of Alzheimer disease (AD), but affected persons are not always aware of this impairment. Unawareness, or anosognosia,^1^ of memory impairment has been associated with increased hours of informal care, greater use of support services, and increased total family care costs.^2,3^ Although anosognosia has been reported as mainly a symptom of late-stage AD,^4^ accumulating evidence suggests that it exists as early as predementia stages^5,6,7,8,9^ (ie, the preclinical and prodromal stages). This later finding may have clinical relevance because it is directly associated with the reliability of a patient's complaints of dysfunction.^10^ Paradoxically, there seems to be a period in which individuals may experience increased awareness of subtle changes in their memory function despite performing well on standardized memory tests (often referred to as subjective cognitive decline [SCD]).^11^ This SCD only takes the participant’s complaints into account, whereas increased awareness compares the participant’s complaints with the informant’s complaints or objective memory function. Of importance, SCD, increased awareness, and reduced awareness in the predementia stage are associated with biomarker and neuroimaging abnormalities consistent with AD pathology^6,7,8,12,13,14,15,16,17,18,19,20^ as well as an increased risk of prospective AD dementia.^8,10,21,22,23^ A consensus is lacking regarding the presence and evolution of altered awareness of memory function across the preclinical and prodromal stages of AD. In particular, the findings of variability of awareness may have implications for the use of SCD in the predementia stages of AD because loss of awareness is associated with reduced validity of the subjective experience of cognitive abilities.

A previous study^24^ found that individuals who are unaware of their cognitive deficits may engage in activities beyond their true functional capacity, thus becoming exposed to potentially dangerous situations. As a consequence, caregivers of patients with anosognosia may experience a greater burden because of the need of increased supervision and control, often leading to early institutionalization.^3^ In contrast, the knowledge or awareness that one is having memory problems is critical to ensure the person’s capability to make healthy and safe everyday life decisions.^25^

To our knowledge, the longitudinal change of awareness of memory function across the earlier AD spectrum is limited to 2 previous studies,^5,9^ both demonstrating that awareness of cognitive impairment decreased and anosognosia appeared approximately 2 to 3 years before dementia onset. Furthermore, previous longitudinal analyses^5^ using the Alzheimer’s Disease Neuroimaging Initiative (ADNI) data found that cognitively normal individuals (both individuals who progressed to having mild cognitive impairment [MCI] and individuals who remained stable after the last study visit) had an overall tendency to complain about their memory. Individuals who progressed to having MCI demonstrated a decrease in awareness of memory function before their MCI diagnosis, indicating that an individual’s self-appraisal of memory function might be affected as AD pathology (eg, amyloid and tau) increases. The findings from these studies call attention to the gap of knowledge we currently have in this area and highlight the importance of investigating awareness and the concurrent association between subject and informant memory complaints across the AD trajectory.

The present study aimed to characterize alterations in awareness of memory function in a large cohort of descendants of individuals from the Colombian kindred with a variant in presenilin (*PSEN1* [OMIM 104311] E280A)^26^ that causes early-onset dementia (estimated median age of dementia onset, 49 years; 95% CI, 49-51 years). Individuals with autosomal dominant AD (ADAD) were studied because penetrance to familial AD is complete and they have a well-characterized disease trajectory from presymptomatic to clinical stages.^26^ Using pseudolongitudinal linear mixed-effects models, we aimed to assess awareness of memory function in individuals carrying the variant compared with awareness in family members who do not carry the variant. We hypothesized that we would observe an initial period of increased awareness in both groups but that memory self-awareness would gradually decrease in the variant carriers, reaching anosognosia before their estimated year of dementia onset.

## Methods

### Design

This cohort study analyzed participants enrolled in the Colombia Alzheimer’s Prevention Initiative Registry.^27^ The Colombia Alzheimer’s Prevention Initiative Registry is an ongoing, longitudinal study that has followed up large families at risk for ADAD since the 1990s.^28^ The registry currently has data from 5800 individuals. The clinical and preclinical courses of this cohort have been well characterized. Variant carriers develop MCI at approximately 44 years of age (95% CI, 43-45 years) and dementia caused by AD at approximately 49 years of age (95% CI, 49-51 years).^26^ For the current cohort study, we analyzed data collected from each participant between January 1, 2000, and July 31, 2019, at the University of Antioquia, Colombia. For all participants, we used the initial completion of their memory complaint scale^29^ as the inclusion criterion. This study was performed in accordance with the principles of the Declaration of Helsinki^30^ and was approved by the ethical research committees of the University of Antioquia in Colombia and Massachusetts General Hospital in Boston. All participants provided written informed consent. All data were deidentified. Participants and researchers were blinded to genetic status. This study followed the Strengthening the Reporting of Observational Studies in Epidemiology (STROBE) reporting guideline.

### Participants

The study sample included 2379 participants, 396 of whom carried the presenilin (*PSEN1* E280A) variant and 1983 of whom were noncarrier family members. All participants had at least 1 parent who carried the *PSEN1* E280A variant but were blinded to their genetic status in accordance with cultural norms and ethical regulations in this community. For the variant carriers, we defined cognitively unimpaired individuals as individuals’ having a Mini-Mental State Examination^31^ score of 26 or greater and a Functional Assessment Staging Test (FAST)^32^ score lower than 3 points. Individuals with cognitive impairment were defined as having FAST scores of 3 or higher. To assess participants’ episodic memory performance, we administered a word list memory test from the Colombian-normed Consortium to Establish a Registry for Alzheimer’s Disease.^33^ The memory test consists of a 10-word list that is repeated 3 times, and individuals are instructed to repeat the list each time they hear it.

### Estimation of Awareness of Memory Function

A memory complaint scale (Spanish version)^29^ was used to estimate self-awareness of memory performance. The memory complaint scale is a 15-item measure that was specifically developed to assess memory complaints in Hispanic older adults. The study partner and self-rated versions of the questionnaire are composed of identical questions framed in the context of current performance. For example, “Do you often forget the first or last names of people you know?” and “Do you have difficulties remembering recent world news or events?” The questions were scored on a 4-point Likert scale (with 0 indicating never; 1, rarely; 2, sometimes; and 3, often). We calculated the sum of these scores for each participant and informant in whom a higher score would indicate more complaints. In addition, we used the discrepancy between the self-rated and the study partner–rated questionnaires to assess awareness of memory function. With this approach, a negative memory awareness index score indicated an overestimation of memory functioning or low memory awareness, meaning that these individuals believed they were functioning at a higher level than their partners rated. In contrast, a positive memory awareness index score indicated underestimation of memory functioning or increased memory awareness, meaning that these individuals believed they were functioning worse than their partners rated. An awareness score of 0 indicated that the participant and the study partner judged memory similarly, suggesting that the participant may have insight into their memory functioning. All participants in the study completed cognitive testing first and the self-reported questionnaires, such as the Memory Complaint Scale and Geriatric Depression Scale, second (at the end of the session).

### Statistical Analysis

All analyses were conducted in Matlab R2017b (MathWorks) and are reported with 2-tailed *P* values (α = .05). Demographic comparisons were made using independent-sample and 2-tailed *t* tests and χ^2^ tests of independence.

We used pseudolongitudinal analyses using linear mixed-effects models to investigate (1) episodic memory (word list learning), (2) participants’ and partners’ complaints, and (3) the awareness index across ages. All models included age as a fixed and random factor. Controlling for sex and educational level did not modify any of the results. Specifically, because this specific variant is almost 100% penetrant for the development of the disease by midlife, we used a previously defined median age at onset for AD dementia for this cohort (49 years) to examine the trajectory of the memory and self-awareness markers of the disease as a function of the carriers’ estimated years to clinical onset.^26^

We evaluated the significance of the intercept at different times (spotlight analysis) and computed a 5000-trial bootstrap to provide 95% CIs around the time at which awareness was significantly nonzero (floodlight analysis). A threshold for reduced memory awareness (anosognosia) was determined as the memory awareness index (the Johnson-Neyman point) at that age when variant carriers reported significantly fewer difficulties than did their study partners. Similarly, a threshold for increased awareness was determined as the awareness index (the Johnson-Neyman point) when variant carriers reported significantly more difficulties than did their partners. To investigate whether episodic memory performance was different between carriers (including impaired and nonimpaired participants) and noncarriers across ages, we conducted a pseudo-longitudinal analysis using linear mixed-effect models that assessed episodic memory (word list learning) with age as a fixed and random factor. To investigate awareness of memory function in the variant and nonvariant carriers, we conducted a pseudo-longitudinal analysis using linear mixed effects models that assessed the self-awareness index for each group with age as a fixed and random factor. We repeated the pseudo–linear mixed effects models in the variant carrier and noncarriers separately to assess participant and study partner reports of complaints with age as a fixed and random factor.

## Results

### Characteristics of the Participants in the Study

The study included 396 variant carriers (mean [SD] age, 32.7 [11.9] years; 200 [50.5%] female), of whom 59 (14.9%) were cognitively impaired, and 1983 cognitively unimpaired noncarriers (mean [SD] age, 33.5 [12.5] years; 1129 [56.9%] female). Table 1 lists the participant characteristics and *P* values for all carriers and noncarriers as well as variant carriers separated into cognitively impaired and unimpaired groups. The carrier cohort included significantly fewer women (200 unimpaired carriers [50.5%] vs 1129 noncarriers [56.9%]; *P* = .02), had lower mean (SD) levels of education (7.8 [4.3] years in carriers vs 8.6 [4.5] years in noncarriers; *P* < .001), had lower Mini-Mental State Examination scores (−1.8 points, *P* < .001), and performed worse on the immediate recall memory task (−1.4 points, *P* < .001) compared with noncarriers. On the memory complaint scale questionnaire, the carriers reported significantly more complaints than noncarriers (1.4 points, *P* = .003). When dividing the carriers into cognitively impaired and unimpaired, the cognitively impaired carriers reported more complaints than the unimpaired carriers (10.1 points, *P* < .001). The cognitively unimpaired carriers reported the same level of complaints as the noncarriers (Table 1). Similarly, the study partners of the variant carriers (both in the unimpaired and impaired cohorts) reported significantly more complaints than the partners of the noncarriers (4.7 points, *P* < .001). When dividing the carriers into cognitively impaired and unimpaired, the partners of the impaired carriers complained significantly more than the partners of the unimpaired carriers (20.8 points, *P* < .001). On the awareness index, awareness was lower in variant carriers than in the noncarriers (−3.3 points, *P* < .001). When dividing the variant carrier cohort into impaired and unimpaired, the impaired carriers had significantly reduced awareness compared with the unimpaired carriers (−10.6 points, *P* < .001).

**Table 1.** Characteristics of the Carriers and Noncarriers of the Presenilin (*PSEN1* E280A) Variant for Autosomal Dominant Alzheimer Disease^a^

Characteristic	Noncarriers (n = 1983)	Unimpaired carriers (n = 337)	*P* value^b^	Impaired carriers (n = 59)	*P* value^c^
Age, y	33.5 (12.5) [18 to 79]	30.0 (10.4) [18 to 60]	<.001	48.5 (6.1) [38 to 72]	<.001
Educational level, y	8.6 (4.5) [0 to 25]	7.8 (4.3) [0 to 20]	<.001	5.3 (4.4) [0 to 19]	<.001
Female, No. (%)	1129 (56.9)	170 (50.5)	.02	30 (50.9)	.95
MMSE score^d^	28.7 (1.9) [15 to 30]	28.1 (2.4) [18 to 30]	<.001	20.1 (5.6) [9 to 30]	<.001
Word list learning^d^	16.9 (4.0) [5 to 30]	15.7 (4.6) [3 to 27]	<.001	7.0 (4.3) [0 to 20]	<.001
Participant complaints^e^	13.7 (8.5) [1 to 45]	13.6 (8.6) [1 to 42]	.93	23.7 (11.2) [2 to 45]	<.001
Partner complaints^e^	9.8 (7.6) [1 to 41]	11.4 (8.4) [1 to 40]	<.001	32.1 (10.2) [1 to 45]	<.001
Awareness index^e^	3.9 (8.4) [−31 to 39]	2.2 (8.8) [−25 to 35]	<.001	−8.4 (14.2) [−36 to 37]	<.001

Abbreviation: MMSE, Mini-Mental State Examination.

^a^ Data are presented as mean (SD) [range] unless otherwise indicated.

^b^ Compared with noncarriers.

^c^ Compared with unimpaired carriers.

^d^ Total score of 30.

^e^ Total score of 45.

### Investigating Episodic Memory Performance Across Age in Carriers and Noncarriers

**Figure 1.**
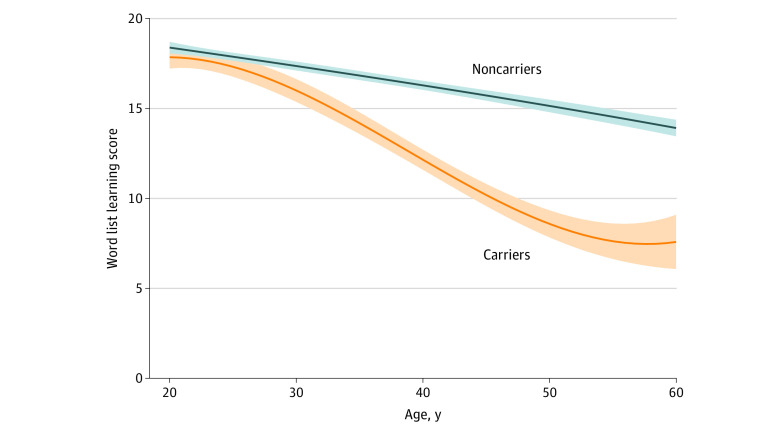
Episodic Memory in Variant Carriers and Noncarriers Shading represents 95% CIs.

In noncarriers, performance on the world list learning significantly decreased with age (mean [SE] estimate, −0.11 [0.01] points per years; *t* = −16.9; *P* < .001) (Figure 1). In the carriers, world list learning decreased compared with the noncarriers (mean [SD] estimate, −0.18 [0.02] points per years; *t* = −10.4; *P* < .001) (Figure 1). Performance on the word list learning test was significantly different between groups from the age of 21.4 years (mean [SD] estimate, −2.5 [0.13] points per years; *t* = −1.96; *P* < .05).

### Evaluating of Memory Self-awareness Across Age in Carriers and Noncarriers

**Figure 2.**
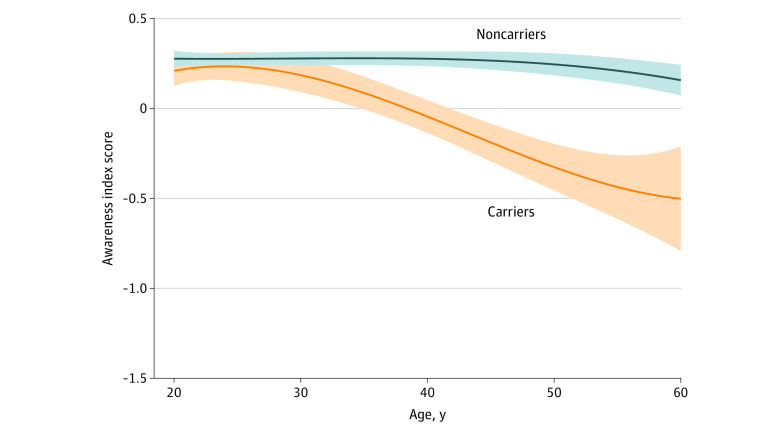
Awareness in Variant Carriers and Noncarriers Negative scores indicate an overestimation of memory functioning or low awareness of memory function, indicating that these individuals believed they were functioning at a higher level than their partners rated. In contrast, a positive awareness index score indicates underestimation of memory functioning or increased awareness of memory function, indicating that these individuals believed they were functioning worse than their partners rated. An awareness score of 0 indicates that the participant and the study partner judged memory similarly, suggesting that the participant had insight into their memory functioning. Shading represents 95% CIs.

In the noncarriers, the awareness index significantly decreased with age (mean [SE] estimate, −0.04 [0.02] discrepant-points per years; *t* = −2.2; *P* = .03) (Figure 2). In the variant carriers, the awareness index decreased compared with the noncarriers (mean [SE] estimate, −0.21 [0.04] discrepant-points per years; *t* = −5.1; *P* < .001) (Figure 2). The awareness index was different between the groups from 22.5-year of age (mean [SE] estimate, −1.1 [0.57] points per years; *t* = −1.96; *P* = .05).

### Investigating Participant and Study Partner Report of Complaints Among the Carriers and Noncarriers

**Figure 3.**
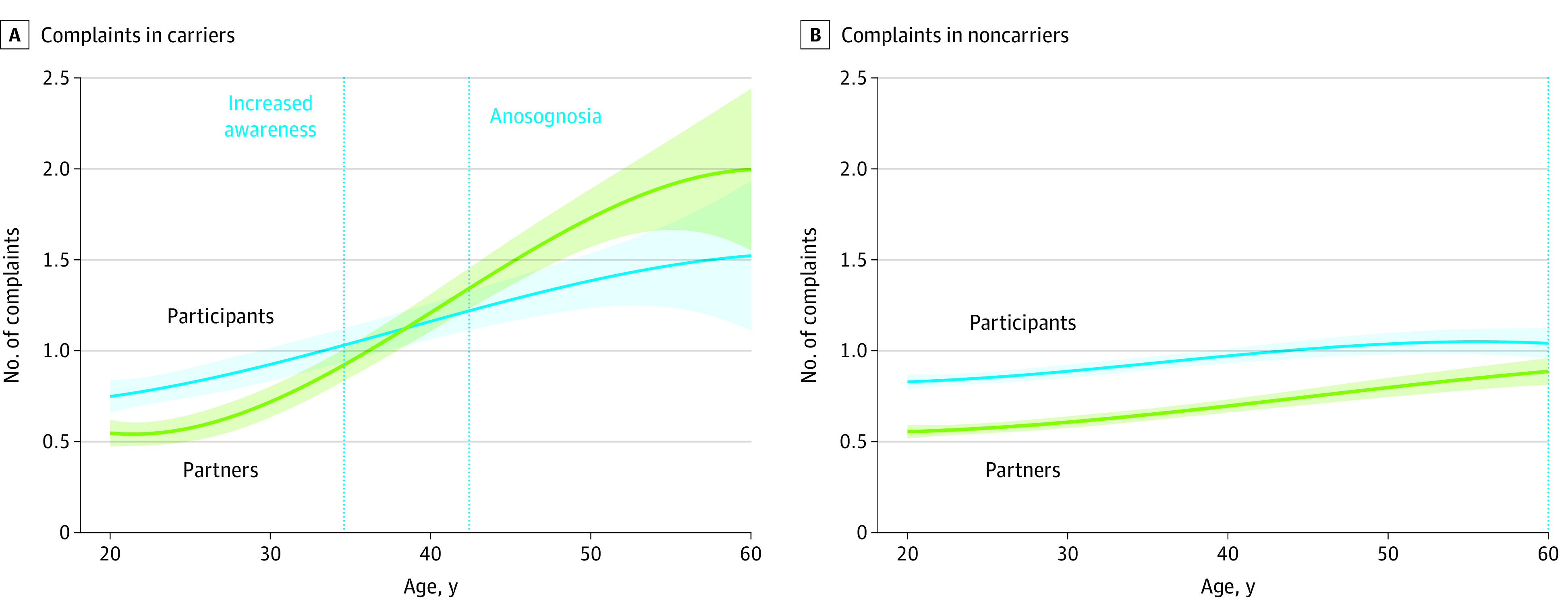
Participant and Partner Complaints Among Variant Carriers and Noncarriers The mean estimated age of dementia onset is 49 years. Shading represents 95% CIs.

The variant carriers complained significantly (increased awareness) more than their study partners until the mean (SD) age of 35.0 (2.0) years and complained significantly less (anosognosia) than their study partner at the mean (SD) age of 43.0 (2.0) years (Figure 3A), 6 years before their estimated median age of dementia onset (49 years; 95% CI, 49-51 years).^26^ Cognitively unimpaired noncarriers complained significantly more (mean [SD], 13.7 [8.5] complaints) than their study partners (mean [SD], 9.8 [7.6] complaints) (increased awareness of memory function) between the ages of 20 and 60 years (10.1 points, *P* < .001) (Figure 3B).

### Investigating the Association Between Complaints and Objective Memory Performance in the Carriers

Across all variant carriers, the Pearson correlation between participant complaints and objective memory performance was 0.46 (*R*^2^ = 0.23), whereas the Pearson correlation between partner complaints and objective memory performance was 0.64 (*R*^2^ = 0.41). When assessing objective memory performance over age with both partner (estimate [SE], −0.0018 [0.00085]; *P* = .03) and participant (estimate [SE], 0.001 [0.00089]; *P* = .26) complaints, only partner complaints were significant (Table 2).

**Table 2.** Association of Partner and Participant Complaints With Memory Performance

Variable	Estimate (SE)^a^	*t*	*P* value
Intercept	9.45 (0.53)	18.5	<.001
Age	−0.10 (0.017)	−6.03	<.001
Partner complaints	0.003 (0.034)	0.095	.93
Participant complaints	−0.076 (0.032)	−2.34	.02
Age and partner complaints	−0.0018 (0.00085)	−2.13	.03
Age and participant complaints	0.001 (0.00089)	1.13	.26

^a^ The *df* was 389 for all.

## Discussion

The present study aimed to characterize alterations in levels of awareness of memory function in a large cohort of individuals who carried a single variant for ADAD compared with family members who did not carry the variant. We observed that increased complaints by the participant were present in both variant and nonvariant carriers 14 years before the estimated median age of dementia onset (49 years; 95% CI, 49-51 years), suggesting that increased awareness of memory function was common and nonspecific in this cohort. In variant carriers only, awareness of memory function decreased in the predementia stages, reaching anosognosia 6 years before dementia onset. These results suggest that discordant self- and informant-reported cognitive decline may provide important information to the practitioner.

Previous research has demonstrated variability of awareness of memory function across the AD spectrum. Although unawareness of memory impairment has predominantly been observed in individuals diagnosed with AD at the dementia stage,^4^ the lack of awareness of memory function has recently been extended to predementia stages of the disease.^5,6,7,8,9,20^ Most of these studies^6,19,20^ have been of cross-sectional cohorts at increased risk by molecular markers, limiting the ability to determine whether an individual’s self-judgment of their own cognitive abilities changed over the course of the disease. However, Wilson et al^9^ found that in a longitudinal cohort of 239 individuals who developed incident dementia, awareness was stable until a mean of 2.6 years (95% credible interval, 22.7 to –1.6) before dementia onset, after which awareness of memory function was shown to decrease rapidly (mean annual change, 20.32; 95% credible interval, –0.37 to –0.28). Similarly, Hanseeuw et al^5^ reported that in 68 individuals who were considered cognitively normal at baseline but later progressed to having MCI, an initial period was observed in which the participant generally reported more difficulties in memory than their study partner, indicating a state of increased awareness followed by a decrease in awareness over time (−0.08 discrepant-points per year). In a subsample of 135 participants with MCI who progressed to having AD dementia during the study, awareness continued to decrease over time (−0.23 discrepant-points per year, *P* < .001).^5^ Subsequently, in these individuals, progressively decreasing awareness and anosognosia (defined as participants reporting significantly fewer difficulties than their study partners) were observed at a mean of 3.2 years (95% CI, 2.8-4.4 years) before progression to dementia.^5^

Our findings are in accordance with these previous findings demonstrating an initial stage in which the variant carriers reported significantly more complaints than their study partners followed by a decrease in memory self-awareness, ultimately reaching anosognosia (defined as the participant complaining significantly less than their study partner) approximately 6 years before their estimated median year of dementia onset. In addition, we observed a slight increase in the number of complaints in both the noncarriers and variant carriers as they approached their estimated year of dementia onset. Compared with noncarriers (eTable in the Supplement), no difference in complaints was found until the mean (SD) age of 35.0 (2.0) years, after which variant carriers had significantly more complaints compared with noncarriers. However, after the age of 52 years, the complaints were no longer significant between variant carriers and noncarriers. These results need to be interpreted in the context of the number of complaints made by the carriers, which started at a sum of complaints of 11.6 at younger than 27 years and ended at 18.6 at older than 52 years (Figure 3A and eTable in the Supplement). These scores correspond to a score of 0 to 1 on the Likert scale (eg, participant reporting never or rarely having difficulties) or a score of 1 to 2 on the Likert scale (eg, participant reporting rarely or sometimes having difficulties). Thus, even though participants in this cohort demonstrated an increase in the number of complaints as they aged, the variant carriers underestimated the severity of these difficulties as the partner’s complaints were increasing further. However, future studies are needed to address the complaints more in detail by using, for example, item analyses, because some items on the questionnaire might be more endorsed than others. Of interest, we found that the partners of variant carriers complained significantly more than did partners of the noncarriers after the age of 28 years (eTable in the Supplement), suggesting that the partner was noticing slight changes in the behavior of the participant before their estimated year of dementia onset. With regard to the awareness index, we observed a positive score in the variant carriers until the age of approximately 43 years (Figure 3 and eTable in the Supplement), after which the awareness index was negative, suggesting the start of anosognosia. In the noncarriers, the awareness index was positive and significantly different from 0 between the ages of 20 to 60 years, suggesting that the noncarriers complained significantly more than their partners (eg, these people believe that they have memory decline despite being in good health).

Furthermore, in additional analyses of the variant carriers only, a significant association was revealed between objective memory performance and participant and partner complaints, suggesting that as memory declined, both the partner and the participant complained more. In addition, objective memory performance decreased before awareness started to decrease in the variant carriers (Figure 1 and Figure 2). However and of more importance, when both sources of complaints were added to the same model, only the partner complaints remained, providing support for the usefulness of also collecting this information from the partner. These results are in line with previous findings^5^ that reflect an underestimation of the participant, likely because of start of anosognosia as the individuals were closer to an AD dementia diagnosis.

There is an increasing yet incomplete understanding of the neurobiological mechanisms that underlie the decrement of self-awareness in AD. Recent neuroimaging findings suggest that anosognosia in AD not only reflects functional alteration in discrete brain regions but also likely results from functional connectivity disruption (as assessed with resting state functional connectivity) between different brain regions (ie, network breakdown).^34,35,36^

These same brain regions are also vulnerable to AD proteinopathies: intracellular neurofibrillary tangles of tau^37,38^ and extracellular amyloid-β peptide proteins.^39,40,41,42,43,44^ There is increasing evidence of an association between anosognosia in the predementia stages with amyloid and tau,^6,9,19,20^ indicating a possible connection among AD pathological deposits, structural brain damage, and functionality of the large-scale brain systems. Furthermore, it has been argued that the disconnection, especially within the self-referential regions, might impair the meta-mnemonic monitoring processes necessary to update the person's knowledge about their memory skills.^45^ The concept of the petrified self suggests that anosognosia is caused by memory deficits that make the sense of self appear as if frozen in time, sometimes reflecting personal features that were accurate in a distant past.^46,47^ Previous longitudinal findings in the ADNI data are in accordance with these concepts, with an observed stable report of subjective complaints over time.^5^ Specifically, the subjective complaints in cognitively normal individuals and patients with MCI that progressed clinically during the study remained relatively stable over time.^5^ Similarly, with the caveat that the current study was pseudo-longitudinal, we observed that the subjective complaints in variant carriers remained stable in relation to age (Figure 3). These findings should also be considered in relation to their episodic memory performance, which decreased over time (mean [SE] estimate, −0.18 [0.02] points per year; *t* = −10.4; *P* < .001) (Figure 1) and which differed significantly from that of the nonvariant carriers from the age of 21.4 years. This apparent frozen state of subjective memory complaints might be attributable to inaccurate evaluations of their change in memory function, such that individuals close to the estimated time of dementia onset remained at the same level of complaints as individuals further from the estimated time of dementia onset.

These findings may have implications for the use of subjective memory complaints in the diagnostic criteria of MCI^10^ as well as using SCD as an inclusion criterion for cognitively normal individuals in clinical trials. Previous studies^48,49,50^ have estimated that the prevalence of memory complaints in the elderly population without dementia ranges from 22% to 60%. Individuals may experience increased awareness of subtle changes in their memory function despite performing well on standardized memory tests for reasons other than AD pathology, including anxiety or fear of potentially developing dementia (nosophobia); psychoaffective disorders, such as neuroticism,^51^ depression,^49,50,52^ and sleep disorders^53^; or normal age-related changes, leading cognitively normal older individuals to complain about their memory more than their partners. In the current study, we found that increased complaints by the participant were observed in both variant carriers and noncarriers, suggesting that increased awareness of memory function was common and nonspecific in this cohort and may be associated with their past experiences of having several family members with dementia (ie, nosophobia). Our results should also be interpreted in the context of this specific cohort. Specifically, all individuals in the study were aware of their high risk of developing AD despite being unaware of their own genetic status. This knowledge may place all participants in this study on high alert for memory problems in themselves and in their relatives as they approach their early to middle 40s. To confirm this, it would have been ideal to compare the carriers and noncarriers studied here with individuals who were unrelated to the kindred, but these data were not available. Nonetheless, this increased awareness of memory function is similar to previous findings in the ADNI cohort demonstrating that cognitively normal individuals and patients with MCI (both participants who progressed clinically and participants who did not progress clinically) had an overall tendency to complain about their memory.^5^ Because of this overlap (ie, nonprogressor participants also complained significantly more than their partners about their memory), increased awareness was not associated with progression in our analyses. With regard to the current findings, individuals in this cohort may have increased vigilance of their own memory function because of the experience of seeing their parents and/or other family members deal with this disease. With regard to the concept of the aforementioned petrified self, our observation of stable complaints among variant carriers (particularly individuals close to their estimated year of dementia onset) may indicate an impairment in the meta-mnemonic monitoring processes necessary to update the person's knowledge about their memory skills. In addition, our findings suggests that some caution is warranted when interpreting subjective reports of individuals at risk for dementia because inaccurate self-estimation of memory impairment may be present.

### Strengths and Limitations

This study has strengths. We had the opportunity to investigate awareness of memory function in one of the largest cohorts of individuals who carry a specific variant for ADAD. Although all participants were blinded to their own genetic status, they all had at least 1 parent who carried the *PSEN1* E280A variant. The clinical and preclinical courses in this cohort have thus been well characterized, and given that the estimated age at onset is approximately 49 years, their younger age also reduces age-related comorbidities often found in sporadic late-onset samples when interpreting memory problems.

This study also has limitations. Although our findings are in accordance with previous longitudinal studies^5,9^ of change in memory self-awareness, we acknowledge that this is a pseudo-longitudinal study. However, as more longitudinal data sets become available, it will be important to investigate whether the findings are similar in those longitudinal samples. The current study assessed awareness based on only memory reports and did not account for other cognitive domains, such as language and executive function. Although not available in the current cohort, future longitudinal studies should investigate the specificity of awareness of memory function compared with other cognitive domains because this may have important clinical relevance. Furthermore, we did not have more specific study partner characteristics because this information was not originally collected for this cohort. However, based on information given from the practitioners collecting the data, most of the partners were spouses (when married) or siblings (when single), potentially influencing the results. For example, it is likely that siblings may experience some anxiety and that this may have impacted their complaint score, or they may carry the genetic variant. However, none of the siblings in the cohort knew their genetic status; thus, the same was true for the siblings of noncarriers and carriers.

The current study did not investigate the association between awareness of memory function to AD pathology, such as amyloid and tau. For instance, in variant carriers, high levels of cortical amyloid burden have been observed at a mean age of 28 years^54^ and elevated regional tau levels in the medial temporal lobe at a mean age of 38 years.^55^ Previous studies^29,56^ have found that self-reported memory complaints in a subset (n = 21) of unimpaired variant carriers of the Colombian kindred were associated with biomarkers of AD pathology. Future longitudinal studies are needed to determine whether an individual’s self-judgment of their own memory abilities changes during the disease and especially as pathological findings increase. By understanding the mechanistic underpinnings of memory self-awareness, we may be able to predict the onset of anosognosia, specifically measured as the loss of self-awareness as objective memory deficits become manifest, because of biomarker evidence of AD pathology.

## Conclusions

In this cohort study, increased participant complaints were observed in both groups, suggesting that increased awareness of memory function was common and nonspecific to AD in this cohort. In variant carriers, awareness of memory function decreased in the predementia stages, reaching anosognosia close to the age of mild cognitive impairment onset, providing support for the usefulness of awareness of memory decline. New biobehavioral preventive research that focuses on identifying individuals in a preclinical state when independent functioning and decision-making are preserved and when strategies to bolster self-awareness may have the greatest impact appears to be needed.

## Notes

Supplement. **eTable.** Memory Complaints and Awareness Scores Split by Age and Mutation Carrier Groups
