# Supplementary material for: Trajectory of Unawareness of Memory Decline in Individuals With Autosomal Dominant Alzheimer Disease
Source: JAMA Netw Open. 2020 Dec 2;3(12):e2027472. doi: 10.1001/jamanetworkopen.2020.27472 (PMC7711319; doi:10.1001/jamanetworkopen.2020.27472)
Supplement: Supplement. — eTable. Memory Complaints and Awareness Scores Split by Age and Mutation Carrier Groups [file jamanetwopen-e2027472-s001.pdf]

Supplementary Online Content

Vannini P, Hanseeuw BJ, Gatchel JR, et al. Trajectory of unawareness of memory decline in individuals with autosomal dominant Alzheimer disease. *JAMA Netw Open*. 2020;3(12):e2027472. doi:10.1001/jamanetworkopen.2020.27472

eTable. Memory Complaints and Awareness Scores Split by Age and Mutation Carrier Groups

This supplementary material has been provided by the authors to give readers additional information about their work.

© 2020 Vannini P et al. *JAMA Network Open.*

eTable. Memory Complaints and Awareness Scores Split by Age and Mutation Carrier Groups

	**Participant Complaints**			**Partners Complaints**			**Awareness Score**		
**Age**	**Non-carriers**	**Carriers**	**p**	**Non-carriers**	**Carriers**	**p**	**Non-carriers**	**Carriers**	**p**
**≤ 27**	12.6 [3, 27]	11.6 [3, 26]	0.08	8.4 [1, 22]	8.6 [2, 21]	0.71	+4.2 [-8, 17] **p<0.001**	+2.9 [-12, 14] **p<0.001**	0.05
**28-35**	13.5 [2, 30]	13.8 [2, 27]	0.72	9.5 [2, 26]	11.5 [2, 25]	**0.04**	+3.9 [-10, 18] **p<0.001**	+2.4 [-15, 17] **p=0.04**	0.16
**36-43**	14.3 [3, 31]	17.7 [3, 38]	**0.007**	9.9 [1, 27]	17.4 [2, 39]	**<0.001**	+4.3 [-12, 19] **p<0.001**	+0.3 [-12, 15] p=0.80	**0.01**
**44-51**	15.7 [3, 35]	21.5 [6, 42]	**0.001**	11.5 [1, 29]	24.8 [3, 40]	**<0.001**	+4.2 [-11, 21] **p<0.001**	-3.3 [-30, 14] **p=0.05**	**<0.001**
**≥ 52**	15.0 [2, 33]	18.6 [3, 39]	0.10	13.1 [1, 28]	27.3 [9, 44]	**<0.001**	+1.9 [-14, 18] **p=0.006**	-8.6 [-34, 12] **p=0.005**	**<0.001**

Participant complaints, partner complaints, and awareness scores are raw scores out of a maximum of 45 points obtained from 15 questions answered on a Likert scale from 0 to 3 (0=never, 1=rarely, 2=sometimes, 3=always). Significant p-values (two-tail p-values<0.05) are highlighted in bold. The 5th and 95th percentiles are under brackets. The p-values in the 'Awareness Score' columns are one-sample t-tests (Does the awareness score differ from 0?).

© 2020 Vannini P et al. *JAMA Network Open.*
